# Pharmacokinetic and Pharmacogenetic Factors Contributing to Platelet Function Recovery After Single Dose of Ticagrelor in Healthy Subjects

**DOI:** 10.3389/fphar.2019.00209

**Published:** 2019-03-18

**Authors:** Qian Zhu, Wanping Zhong, Xipei Wang, Liping Mai, Guodong He, Jiyan Chen, Lan Tang, Shuwen Liu, Weihua Lai, Shilong Zhong

**Affiliations:** ^1^Guangdong Provincial Key Laboratory of Coronary Heart Disease Prevention, Guangdong Cardiovascular Institute, Guangdong Provincial People's Hospital, Guangdong Academy of Medical Sciences, Guangzhou, China; ^2^Department of Pharmacy of Guangdong Provincial People's Hospital, Guangdong Provincial People's Hospital, Guangdong Academy of Medical Sciences, Guangzhou, China; ^3^School of Pharmaceutical Sciences, Southern Medical University, Guangzhou, China

**Keywords:** genetic variants, healthy subjects, pharmacokinetics, recovery of platelet function, ticagrelor

## Abstract

**Objectives:** This study aimed to elucidate the contribution of candidate single nucleotide polymorphisms (SNPs) related to pharmacokinetics on the recovery of platelet function after single dose of ticagrelor was orally administered to healthy Chinese subjects.

**Methods:** The pharmacokinetic profiles of ticagrelor and its metabolite AR-C124910XX (M8), and the platelet aggregation (PA), were assessed after 180 mg of single-dose ticagrelor was orally administered to 51 healthy Chinese subjects. Effects of CYP2C19^*^2, CYP2C19^*^3, CYP3A5^*^3, UGT1A1^*^6, UGT1A1^*^28, UGT2B7^*^2, UGT2B7^*^3, SLCO1B1 388A>G, and *SLCO1B1* 521T>C, on the pharmacokinetics of ticagrelor and M8, and platelet function recovery were investigated.

**Results:** The time to recover 50% of the maximum drug effect (RT_50_) ranging from 36 to 126 h with 46.9% CV had a remarkable individual difference and was positively associated with the half-life (t_1/2_) of M8 (*r* = 0.3901, *P* = 0.0067). The time of peak concentration (T_max_) of ticagrelor for CYP2C19^*^3 GG homozygotes was significantly higher than that of GA heterozygotes (*P* = 0.0027, *FDR* = 0.0243). Decreased peak concentration (C_max_) of M8 was significantly associated with *SLCO1B1* 388A>G A allele (*P* = 0.0152, *FDR* = 0.1368). CYP2C19^*^2 A was significantly related to decreased C_max_ of M8 (*P* = 0.0455, *FDR* = 0.2048). While, the influence of these nine SNPs on the recovery of platelet function was not significant.

**Conclusion:** Our study suggests that the elimination of M8 is an important factor in determining the recovery of platelet function. Although CYP2C19 and SLCO1B1 genetic variants were related to the pharmacokinetics of ticagrelor or M8, they did not show a significant effect on the platelet function recovery in this study.

**Clinical Trial Registration:** https://clinicaltrials.gov/ct2/show/NCT03092076, identifier: NCT03092076

## Introduction

Ticagrelor, a member of a novel chemical class of antiplatelets, is the first reversibly binding, noncompetitive, orally bioavailable P2Y12 receptor antagonist that acts directly without requiring metabolic activation (James et al., 2009; Teng et al., 2010; Wiviott and Steg, 2015). Compared with clopidogrel, ticagrelor can provide more potent and consistent antiplatelet effects with faster onset and offset (Gurbel et al., 2009). Ticagrelor is recommended as an antiplatelet therapy for patients with acute coronary syndrome (ACS) according to several guidelines (Wallentin et al., 2009; Levine et al., 2016). Ticagrelor is a relatively powerful and safe antiplatelet agent that remarkably reduces the composite outcome of cardiovascular death, myocardial infarction, or stroke but increases the risk of non-coronary artery bypass grafting (CABG)-related major bleeding (Wallentin et al., 2009). Patients treated with ticagrelor awaiting CABG are associated with an increased risk of perioperative bleeding complications (Hansson et al., 2016).

This issue is important for patients who need urgent surgical procedures, such as CABG. The antiplatelet efficacy of ticagrelor must be balanced with the risk of bleeding. Therefore, the timing of the last dose of ticagrelor should be cautious. In this context, the protocol of a PLATO trial recommends withholding ticagrelor/placebo for 24– 72 h preoperatively (Wallentin et al., 2009). According to the United States label of ticagrelor and the European Society of Cardiology guidelines on non-ST-segment elevation ACS, ticagrelor treatment should be administered at least 5 days before surgery to allow the adequate recovery of platelet function. However, a PLATO CABG substudy (De Waha et al., 2016) and another study (Tomšič et al., 2016) have suggested that the interval can be shortened to 2–3 days. Shortened discontinuation interval before surgery, which may increase the bleeding risk, and the optimal time of terminating the ticagrelor treatment before cardiac surgery is controversial.

The timing of terminating the ticagrelor treatment is primarily based on the sufficient recovery of platelet function after drug discontinuation, and platelet function recovery is acceptable after 72 h in most patients but with a considerable interindividual variability (Hansson et al., 2016). The platelet inhibitory effects of ticagrelor are predominantly determined by the plasma exposures of ticagrelor and the pharmacologically equipotent metabolite M8 (AR-C124910XX) metabolized by cytochrome P450 3A (CYP3A) (Storey et al., 2007; Gurbel et al., 2009; Zhou et al., 2011). Genetic variants related to metabolic enzymes are key factors in drug metabolism and disposition, potentially leading to interindividual variation in platelet inhibitory responses. Varenhorst et al. (2015) reported that three single nucleotide polymorphisms (SNPs), such as CYP3A4, UGT2B7, and SLCO1B1, affect ticagrelor levels in a large cohort of Caucasian patients with ACS but do not translate into any detectable effect on the efficacy or safety of ticagrelor. However, the correlation between the pharmacokinetics and recovery of platelet activity after a single dose of ticagrelor in Chinese people and their genetically influential factors has yet to be investigated.

A study was conducted among 51 healthy Chinese volunteers after they received a single oral dose of ticagrelor (180 mg) to identify the pharmacokinetic and pharmacogenetic factors that contribute to the recovery of platelet activity. This study provided a clinical basis for administering individualized dosage regimens and determining the optimal time of terminating the ticagrelor treatment before coronary surgery to improve efficacy and reduce adverse events.

## Methods

### Study Population and Design

A single-dose, open-label, and prospective study on ticagrelor was carried out at a single center. Each subject in the study was required to have normal physical examination, vital signs, electrocardiogram, and clinical laboratory test results, but exceptions were given if an abnormal value was not clinically significant. Subjects were excluded if they had tobacco or alcohol abuse; suffered from any conditions known to interfere with drug absorption, distribution, metabolism, or excretion; exhibited intolerance or hypersensitivity to drugs with mechanisms similar to that of ticagrelor; had a history of coagulation disorders; received any drugs within half a month before enrollment; participated in a clinical study in the past 3 months; and donated blood within 1 month prior to the study. Written informed consent was obtained from all participants. The study was conducted in compliance with the guidelines of the Declaration of Helsinki and approved by the Research Ethics Committee of Guangdong General Hospital.

A total of 51 healthy Chinese volunteers were enrolled and given a single oral dose of ticagrelor (180 mg) with 100 mL of water after they fasted overnight. Any causally related adverse events were monitored. The pharmacokinetic data and correlation with platelet function recovery of ticagrelor and M8 were reported and evaluated on the basis of the principle of population pharmacodynamics (PPD). Nine candidate SNPs, namely, CYP2C19^*^2 (681G>A, rs4244285), CYP2C19^*^3 (636G>A, rs4986893), CYP3A5^*^3 (6986A>G, rs776746), UGT1A1^*^6 (211G>A, rs4148323), UGT1A1^*^28 ((TA)6>(TA)7, rs8175347), UGT2B7^*^2 (802T>C, rs7439366), UGT2B7^*^3 (211G>T, rs12233719), *SLCO1B1* 388A>G (rs2306283), and *SLCO1B1* 521T>C (rs4149056), were selected to determine the genetic variants that influence the recovery of platelet function by modulating the pharmacokinetic mechanism of ticagrelor. This study was registered at ClinicalTrials.gov (NCT03092076).

### Pharmacokinetics

To determine ticagrelor and its metabolites, blood samples were collected in EDTA-anticoagulated tubes before dosing and at 0.5, 1, 1.5, 2, 3, 4, 6, 8, 12, 24, and 48 h after dosing and then centrifuged at 3,000 rpm for 10 min at 4°C. The blood cells and plasma were individually transferred to storage tubes and stored at −80°C for analysis. High-performance liquid chromatography coupled with tandem mass spectrometry (LC-MS/MS) assay was developed and validated for the simultaneous determination of ticagrelor and its metabolites in human plasma as previously described (Zhong et al., 2016). Details are provided under Supplementary Materials.

The pharmacokinetic parameters of ticagrelor and M8 were estimated for each subject by using a noncompartmental analysis function in Phoenix WinNonlin version 6.3 (Pharsight, Cary, NC, USA). The peak concentration (C_max_) and time of peak concentration (T_max_) were directly estimated from the concentration–time profile. Elimination half-life (t_1/2_) was calculated by ln2/λ_z_, where λ_z_ is the first-order rate constant associated with the terminal (log-linear) portion of the curve. Linear trapezoidal calculation method was selected to calculate the area under curve (AUC).

### Platelet Function Testing

Basic PPD was applied to evaluate the antiplatelet effects of ticagrelor. For platelet function tests, whole blood samples (2 × 2 mL) were collected with BD Vacutainer sodium citrate tubes (1:9) at the following time points (number of volunteers): 0.5 (22), 1 (25), 2 (47), 4 (16), 8 (15) and 24 h (16) and 2 (16), 3 (14), 3.29 (1), 5 (16), 6 (5), 7 (23), 7.04 (1), 9 (4), 10 (3), 11 (4), 23 (3), and 24 days (3) after administration. ADP-stimulated PA was measured within 2 h of sampling through light transmittance aggregometry with 20 μmol/L ADP as an agonist on Chrono-log PA Systems (Vastec Medical. Ltd.). The PA post-dose until recovery to the baseline was expressed in percentage.

A decentralized sampling design was used in platelet function testing. Thus, the missing PA data were imputed through Bayesian simulation. First, the data on the maximal drug effect to full recovery to the baseline PA were included in model development. The independent variable of the recovery model of PA was time, considering that antiplatelet effects were slightly related to drug concentration. A sigmoid maximal effect model (Equation 1) was used to fit the observed PA data and simulate the missing ones by using NONMEM 7.2.0 (Icon Development Solutions, Ellicott City, MD, USA).

(1) $$\begin{array}{r} \text{PA}={\text{E}}_{\text{max}}+{\text{R}}_{\text{max}}\cdot {\text{t}}^{\text{γ}}/\left({\text{RT}}_{50}^{\text{γ}}+{\text{t}}^{\text{γ}}\right) \end{array}$$

where E_max_ is the maximum drug effect, R_max_ is the maximal recovery of PA, RT_50_ is the time to recover 50% of the maximum drug effect, γ is the sigmoidicity or shape factor, and t is time in hours. Second, 1,000 simulations were performed based on the sigmoid model, and the missing data were imputed with the simulated median at each time point by using R (version 3.2.4). RT_50_ and recovery day to the baseline PA (RECDAY) were set to represent the recovery of platelet effect. RECDAY was defined as the time to recover to the baseline PA in days in accordance with the standard from the clinical laboratory of our hospital (ADP-induced PA = 69%).

### Genotyping for Candidate SNPs

Genomic DNA was isolated from blood by using a TIANamp genomic DNA kit (Tiangen Biotech Co. Ltd., Beijing, China). The quality and quantity of the DNA were assessed using a NanoDrop 2000 spectrophotometer (Thermo Scientific, USA). DNA samples were genotyped for the candidate SNPs, namely, CYP2C19^*^2, CYP2C19^*^3, CYP3A5^*^3, UGT1A1^*^6, UGT1A1^*^28, UGT2B7^*^2, UGT2B7^*^3, SLCO1B1 388A>G, and *SLCO1B1* 521T>C, through allelic discrimination with a TaqMan SNP assay by using an ABI Vii7 real-time PCR system (Applied Biosystems, USA).

TaqMan genotyping was performed in a PCR system with a total volume of 10 μL containing 5 μL of 2 × TaqMan Genotyping Master Mix, 20 × TaqMan primer/probe mix, 20 ng of DNA, and RNase-free water. The following thermocycling conditions were used for PCR: an initial denaturation at 95°C for 10 min, followed by 40 cycles of denaturation at 95°C for 15 s, and extension at 60°C for 1 min. The sequences of primers and probes for genotyping are shown under (Table S1).

### Statistical Analysis

Demographic and clinical characteristics were summarized using counts (percentages) for categorical variables and mean ± SD for continuous variables. The continuous variables with normal distribution were analyzed via a Kolmogorov–Smirnov goodness-of-fit test. If the ranges of the variables were skewed, logarithmic transformation was performed prior to analysis. The clinical variables were compared through repeated measures ANOVA before and after ticagrelor was administered. The categorical variables were compared through a χ^2^-test or a Fisher's exact test as appropriate. Spearman correlation coefficients were calculated to describe the correlation between baseline clinical characteristics, pharmacokinetic parameters, and platelet function recovery after a single dose of ticagrelor was administered. Similarly, the correlation between pharmacokinetics and the recovery of platelet function to ticagrelor was determined. A Mann–Whitney *U*-test was conducted to compare the two groups, and a Kruskal–Wallis test was performed to compare multiple groups. Results with *P* < 0.05 were considered statistically significant. Statistically significant raw *P*-values were adjusted for multiple comparisons to control the False Discovery Rate (*FDR*), *FDR* threshold here was set at 0.20 due to it is a rigorous full-time-point pharmacokinetic–pharmacodynamic (PK–PD) study and exploringly pharmacogenetic study. Data analyses were performed on IBM SPSS 21.0 (IBM SPSS Inc., Chicago, IL, USA).

## Results

### Clinical Characteristics and Their Effects on the Pharmacokinetic Parameters and Recovery of Platelet Function

A total of 51 healthy Chinese participants completed the study. They tolerated the single oral dose of ticagrelor (180 mg). No adverse events and clinically significant changes were observed in physical examination, vital signs, and clinical laboratory test results at 7 days post-dose were reported. Table 1 shows the baseline clinical characteristics at pre-dose compared with 7 days post-dose and their effects on the pharmacokinetics of ticagrelor and the recovery of platelet function. Of the 51 participants with a mean age of 24.5 ± 2.3 years, 52.9% were males, and BMI was 20.7 ± 2.3 kg/m^2^.

**Table 1:** Baseline clinical characteristics and their effects on pharmacokinetics and the recovery of platelet function in 51 healthy subjects.

**Variable**	***N* (%) or Mean ± SD**	**Comparison with 7 days post-dose^a^**	***P*****-value of ticagrelor**				***P*****-value of M8**				**RT_**50**_^†^**	**RECDAY^^†^^**
			**t_**1/2**_**	**T_**max**_**	**C_**max**_**	**AUC**	**t_**1/2**_**	**T_**max**_**	**C_**max**_**	**AUC**		
Age (years)	24.51 ± 2.32	/	0.632	0.748	0.137	0.986	0.163	0.366	0.107	0.675	0.109	0.012
Male	27 (52.94%)	/	0.004	0.010	0.122	0.865	0.497	0.034	0.007	<0.001	0.149	0.014
BMI, kg/m^2^	20.69 ± 2.32	/	0.986	0.805	0.079	0.696	0.358	0.757	0.125	0.062	0.130	0.108
**LABORATORY CHARACTERISTICS**												
FIB, g/L	2.74 ± 0.46	0.880	0.206	0.072	0.219	0.165	0.345	0.198	0.060	0.003	0.702	0.469
APTT, sec	38.24 ± 3.69	0.108	0.346	0.838	0.900	0.859	0.577	0.798	0.735	0.208	0.750	0.284
TT, sec	16.34 ± 0.90	0.880	0.079	0.788	0.601	0.526	0.769	0.042	0.788	0.012	0.551	0.206
PT-A, %	98.29 ± 8.68	0.018	0.209	0.374	0.624	0.191	0.253	0.505	0.245	0.166	0.989	0.754
PT, sec	13.3 ± 0.46	<0.001	0.531	0.502	0.864	0.402	0.456	0.456	0.190	0.124	0.369	0.643
INR	1.01± 0.05	<0.001	0.459	0.209	0.734	0.349	0.978	0.560	0.814	0.204	0.206	0.983
WBC, 10^∧^9/L	6.05 ± 1.06	0.075	0.472	0.692	0.167	0.692	0.580	0.506	0.150	0.458	0.675	0.835
RBC^1^, 10^∧^9/L	4.86 ± 0.62	0.261	0.069	0.155	0.174	0.953	0.420	0.116	0.014	<0.001	0.345	0.353
MCH, pg	29.30 ± 2.16	0.698	0.817	0.768	0.609	0.493	0.626	0.394	0.747	0.449	0.397	0.921
PLT, 10^∧^9/L	229.49 ± 47.84	0.154	0.483	0.758	0.923	0.356	0.937	0.324	0.751	0.502	0.269	0.414
URIC, umol/L	342.81 ± 76.14	0.305	0.004	0.029	0.996	0.054	0.529	0.778	0.121	0.036	0.198	0.036
CREA, umol/L	71.13 ± 13.97	0.007	0.064	0.030	0.009	0.058	0.960	0.144	0.055	<0.001	0.988	0.326
ALT, U/L	17.33 ± 9.86	0.569	0.312	0.791	0.011	0.589	0.414	0.327	0.003	0.007	0.794	0.923
TP, g/L	73.46 ± 5.01	0.164	0.985	0.998	0.975	0.768	0.682	0.975	0.312	0.560	0.558	0.066
ALB, g/L	46.01 ± 2.96	0.205	0.756	0.457	0.127	0.427	0.567	0.835	0.071	0.407	0.754	0.004
DBIL, umol/L	4.48 ± 0.92	0.502	0.806	0.328	0.168	0.170	0.136	0.388	0.286	0.003	0.576	0.860
TBIL, umol/L	15.80 ± 5.51	0.004	0.578	0.386	0.201	0.500	0.691	0.975	0.073	0.018	0.574	0.590
Heart rate, bpm	67.51 ± 9.31	0.873	0.302	0.580	0.304	0.425	0.008	0.020	0.491	0.331	0.365	0.385
T interval, ms	408.43 ± 25.45	0.379	0.226	0.908	0.368	0.718	0.046	0.039	0.378	0.393	0.660	0.326
ADP, %	68.18 ± 6.35	/	0.439	0.724	0.941	0.829	0.710	0.950	0.938	0.350	0.154	0.038
ERY^b^	6 (11.76%)	/	0.952	0.339	0.244	0.492	0.720	0.731	0.227	0.698	0.453	0.576
RBC^2^	–	/	/	/	/	/	/	/	/	/	/	/
OB	–	/	/	/	/	/	/	/	/	/	/	/

*BMI, body mass index; FIB, fibrinogen; APTT, activated partial thromboplastin time; TT, thrombin time; PT-A, prothrombin activity; PT, prothrombin time; INR, international normalized ratio; WBC, white blood cell count; RBC^1^, red blood cell count; MCH, mean corpuscular hemoglobin; PLT, platelet count; URIC, uric acid; CREA, creatinine; ALT, alanine aminotransferase; TP, total protein; ALB, albumin; DBIL, direct bilirubin; TBIL, total bilirubin; ADP, RBC^2^, red blood cell in urine; ERY, erythrocyte; OB, stool occult blood test; C _max_, Peak concentration; T _max_, time of peak concentration; t_1/2_, elimination half-life; AUC, area of plasma concentration–time curve; RT_50_, time to 50% recovery of maximum drug effect; RECDAY, recover day to the baseline PA. All correlation analyses were undertaken using Spearman correlation coefficients except those variables marked a, b*.

a *Repeated measures data ANOVA*.

b *Mann-Whitney U-test*.

† *RT_50_ and RECDAY were successfully acquired in 47 subjects*.

*The color values refer to the results with P < 0.05 that considered as significant*.

### Pharmacokinetic Analysis

**Figure 1:**
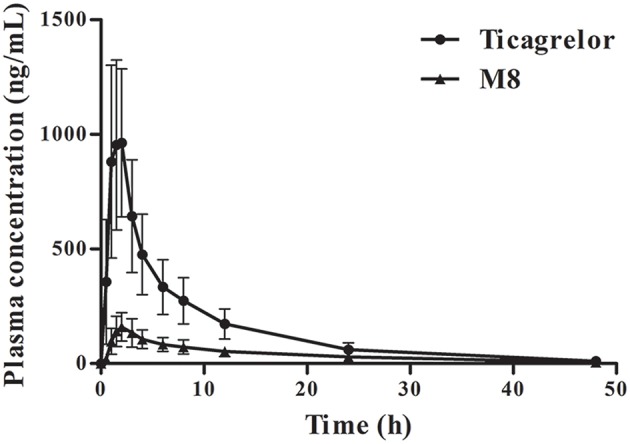
Pharmacokinetic profiles of ticagrelor and M8 in 51 healthy subjects.

The plasma concentration–time profiles of ticagrelor and its metabolite M8 obtained in the 51 subjects administered with a single dose of 180 mg of ticagrelor are presented in Figure 1. The pharmacokinetic parameters of ticagrelor and M8 are summarized in Table 2. The absorption of ticagrelor from the gastrointestinal tract was rapid, but it widely varied with T_max_ of 0.5–4 h (mean ± SD: 1.7 ± 0.7 h) and C_max_ of 494.3–1,929 ng/mL (1,129 ± 366.0 ng/mL), leading to a wide variation in its systemic exposure, with AUC_0−t_ of 3,558–1,1693 ng·h/mL (7,276 ± 2,174 ng·h/mL). The pharmacokinetics of M8 included T_max_ of 1–6 h (2.7 ± 1.0 h), C_max_ of 75.3–427 ng/mL (170.3 ± 81.1 ng/mL) and AUC_0−t_ of 877.1–4,336 ng·h/mL (2,094 ± 754.3 ng·h/mL). The AUC ratio of M8 to ticagrelor was 0.31 ± 0.15, and the C_max_ ratio was 0.16 ± 0.06. Ticagrelor and M8 had plasma t_1/2_ of 6.2–17.0 h (8.5 ± 1.6 h) and 8.0–102.1 h (21.9 ± 20.6 h), respectively.

**Table 2:** Pharmacokinetic parameters for ticagrelor and M8 in 51 healthy subjects after a single oral dose of 180 mg of ticagrelor (Mean ± SD).

**Parameter**	**Ticagrelor**	**M8**
C_max_ (ng/mL)	1,129.28 ± 3,66.02	170.34 ± 81.05
T_max_ (h)	1.68 ± 0.68	2.65 ± 1.04
t_1/2_ (h)	8.51 ± 1.63	21.87 ± 20.57
AUC_0−t_ (ng·h/mL)	7,276.09 ± 2,173.62	2,093.50 ± 754.33

*Data are presented as mean ± SD. C_max_, peak concentration; T_max_, time of peak concentration; t_1/2_, elimination half-life; AUC_0−t_, area of plasma concentration–time curve*.

### Platelet Response to Ticagrelor Treatment

**Figure 2:**
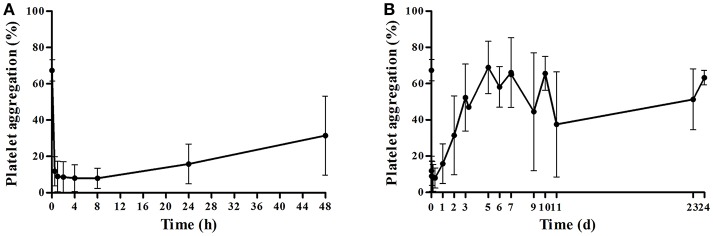
Average platelet aggregation after a single dose of ticagrelor in healthy subjects. **(A)** Average platelet aggregation during 0–48 h after a single 180 mg oral dose of ticagrelor (mean ± SD); **(B)** average platelet aggregation within all points of time after a single 180 mg oral dose of ticagrelor (mean ± SD).

**Figure 3:**
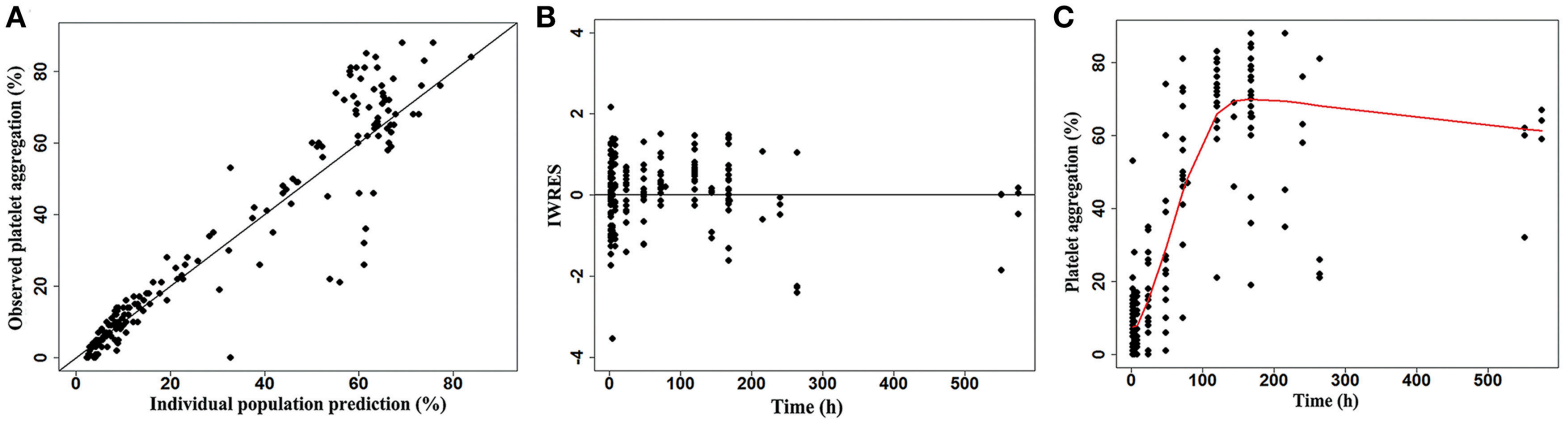
Sigmoid maximal effect model of platelet aggregation. Goodness of fit plots: **(A)** Observed vs. individual population predicted values; **(B)** individual weighted residuals vs. time; **(C)** scatter plot of time vs. platelet aggregation, and the prediction curve.

Platelet response was successfully tested in 47 of the 51 volunteers. The observed PA showed great individual difference after treatment with 180 mg of ticagrelor (Figure 2). The PA of all the participants quickly declined and reached 93.3% of the maximum platelet inhibition within an average time of 0.5 h post-dose. The measured antiplatelet effects had modest individual variability before 8 h at which they reached a maximum platelet inhibition of 88.2%. However, PA gradually recovered to the baseline PA with variably different recovery periods. The population estimates of E_max_ (CV%), R_max_, RT_50_, and γ were 6.81% (14%), 58.7% (4%), 54.8 h (9%), and 3.15 (11%) in the sigmoid maximal effect model, respectively. The interindividual variabilities of E_max_, RT_50_, and R_max_ were 67%, 50.3%, and fixed 0, respectively. The proportional residual error was 22.5%. The individual whole profile of the PA recovery could then be obtained through 1,000 simulations based on this model (Figure 3). A total of 565 imputed data were compared, and the simulated median was compared with 187 observed data. Finally, the estimated RT_50_ and RECDAY of 47 subjects ranged from 27.27 h to 166.94 h (mean ± SD: 60.43 ± 28.35 h) and from 2 days to 24 days (13.21 ± 8.65 days), respectively. The ADPs of all subjects were monitored until the ADP-induced PA reach to 69%, leading to an increase in the average RECDAY to 13 days. The median was 10 days. Overall, the recovery of the platelet function of the 47 participants with a single-dose ticagrelor showed a dramatic interindividual variability.

### Effects of Pharmacokinetic Factors on the Recovery of Platelet Function

**Figure 4:**
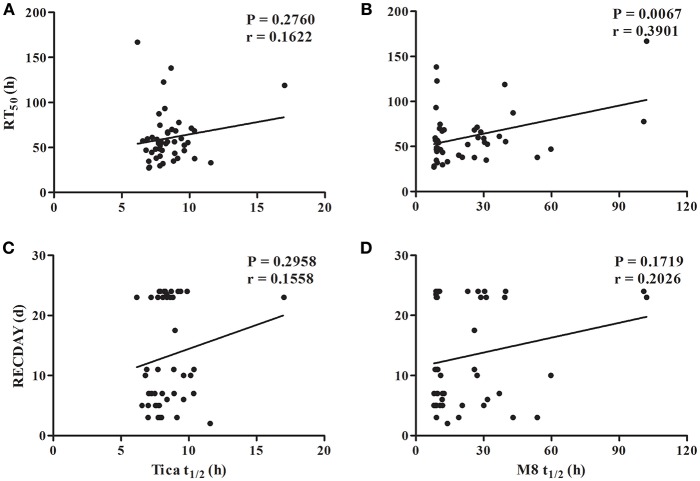
Association of half-life of ticagrelor and M8 with recovery of platelet function. **(A)** Association of half-life (t_1/2_) of ticagrelor with the time to recover 50% of the maximum drug effect (RT_50_); **(B)** association of t_1/2_ of M8 with RT_50_; **(C)** association of t_1/2_ of ticagrelor with the recovery day to the baseline platelet aggregation (RECDAY); **(D)** association of t_1/2_ of M8 with the RECDAY.

To elucidate the pharmacokinetic factors that contribute to the interindividual variation in the recovery of platelet function, and to provide insights into whether the pharmacokinetic profile of ticagrelor could serve as a basis for determining the recovery of platelet function, we assessed whether the pharmacokinetics of ticagrelor was correlated with the recovery of platelet functions (RT_50_ and RECDAY). Results revealed that t_1/2_ of M8 was significantly and positively associated with RT_50_ with Spearman correlation coefficients of 0.3901 (*P* = 0.0067, Figure 4), but the associations between t_1/2_ of M8 and RECDAY and between the elimination of the parent drug ticagrelor and RECDAY were not significant (*P* > 0.05). Overall, RT_50_ was prolonged if the major active metabolite M8 was eliminated gradually as t_1/2_ of M8 was lengthened regardless of the elimination of ticagrelor.

### Effects of Genotypes on Pharmacokinetic Parameters

To investigate the influence of genetic variants related to ticagrelor metabolism on the variability of the pharmacokinetics of ticagrelor and platelet response, we selected nine candidate SNPs in *CYP2C19, CYP3A5, UGT1A1, UGT2B7*, and *SLCO1B1*, which have a relatively high variant allele frequency in Chinese and have a role in the pharmacokinetic process (Jada et al., 2007; Kim et al., 2008; Wallentin et al., 2010; Wang et al., 2012; Lu et al., 2015; Varenhorst et al., 2015).

The association between the candidate SNP genotypes and the pharmacokinetics of ticagrelor and M8 is presented in Table 3. The mutant allele CYP2C19^*^2 A was associated with decreased C_max_ (*P* = 0.0455, *FDR* = 0.2048) of M8. The mean C_max_ of M8 was successively reduced in wildtype (n = 27), heterozygous (n = 16) and homozygous (n = 8) mutant individuals with values of 179.6 ± 70.1, 178.4 ± 105.9, and 122.7 ± 39.4 ng/mL, respectively. The mean T_max_ of M8 in CYP2C19^*^2 AA heterozygous (3.00 ± 0.76 h) and GA homozygous (3.09 ± 1.44 h) mutant individuals was longer than that in the GG wildtype (2.28 ± 0.66 h, *P* = 0.0296), but the FDR was >0.20. T_max_ of ticagrelor for CYP2C19^*^3 GG homozygotes (*n* = 42) was significantly 38.3% higher than those for GA heterozygotes (*n* = 9) of CYP2C19^*^3 (1.80 ± 0.67 h vs. 1.11 ± 0.42 h, respectively; *P* = 0.0027, *FDR* = 0.0243). For *SLCO1B1* 388A>G, only three subjects exhibited the AA genotype, so we merged them with those with the GA genotypes (*n* = 19) into the A allele group (*n* = 22) for analysis. *SLCO1B1* 388A>G A allele carriers had ~39.0% lower C_max_ (138.8 ± 55.35 vs. 193.0 ± 90.90 ng/mL, respectively; *P* = 0.0152, *FDR* = 0.1368) and 31.0% higher t_1/2_ (26.6 ± 22.3 vs. 18.3 ± 18.8 h, respectively; *P* = 0.0373, *FDR* = 0.3357) of M8 compared with those of the *SLCO1B1* 388A>G GG carriers (*n* = 29). But, the FDR for the association between SLCO1B1 388A>G with t_1/2_ was not < 0.20, probably because of the small frequencies in the SLCO1B1 388A>G AA group. The CYP3A5^*^3, UGT1A1^*^28, UGT2B7, and *SLCO1B1* 521T>C genotypes had no statistically significant effect on the pharmacokinetic parameters of ticagrelor and M8.

**Table 3:** Association of candidate SNPs with pharmacokinetics and the recovery of platelet function after a single 180 mg oral dose of ticagrelor.

**SNP**	**Ticagrelor**				**M8**				**RT${\text{RT}}_{50}^{†}$ (h)**	**RECDAY^†^ (d)**
	**C_**max**_ (ng/mL)**	**T_**max**_ (h)**	**t_**1/2**_(h)**	**AUC (ng·h/mL)**	**C_**max**_ (ng/mL)**	**T_**max**_ (h)**	**t_**1/2**_(h)**	**AUC (ng·h/mL)**		
CYP2C19*2										
GG (*n* = 27)	1,189 ± 367.1	1.56 ± 0.63	8.80 ± 1.98	7,369 ± 2,087	179.6 ± 70.09	2.28 ± 0.66	16.74 ± 10.69	2,097 ± 841.7	59.83 ± 4.62	15.25 ± 1.78
GA (*n* = 16)	1,056 ± 310.8	1.75 ± 0.84	8.18 ± 1.14	7,325 ± 2,451	178.4 ± 105.9	3.09 ± 1.44	23.27 ± 24.73	2,191 ± 719.4	51.67 ± 4.40	9.97 ± 1.95
AA (*n* = 8)	1,073 ± 468.7	1.94 ± 0.50	8.21 ± 1.06	6,865 ± 2,114	122.7 ± 39.35	3.00 ± 0.76	36.39 ± 30.66	1887 ± 503.4	78.62 ± 17.60	13.19 ± 3.38
*P-value*	0.5818	0.2579	0.4472	0.9246	0.0455	0.0296	0.2095	0.7606	0.4860	0.2460
FDR	0.7574	0.5803	0.8050	0.9246	0.2048	0.2664	0.4714	0.7606	0.8748	0.7380
CYP2C19*3										
GG (*n* = 42)	1,135 ± 361.3	1.80 ± 0.67	8.48 ± 1.10	7,390 ± 2,207	168.3 ± 76.56	2.67 ± 0.97	21.86 ± 21.33	2,070 ± 752.8	60.57 ± 4.68	13.23 ± 1.41
GA *(n = 9)*	1,105 ± 409.2	1.11 ± 0.42	8.68 ± 3.24	6,746 ± 2,043	179.6 ± 104.4	2.56 ± 1.40	21.93 ± 17.66	2,203 ± 797.3	59.71 ± 9.03	13.13 ± 3.03
*P-value*	0.7574	0.0027	0.2213	0.4220	0.9409	0.3511	0.9704	0.6476	0.9550	0.7860
FDR	0.7574	0.0243	0.7239	0.6916	0.9828	0.6320	0.9704	0.7606	0.9550	0.9800
CYP3A5*3										
*1/*1 or *1/*3 (*n* = 29)	1,154 ± 352.4	1.64 ± 0.69	8.52 ± 1.94	7,202 ± 2,201	172.8 ± 82.53	2.69 ± 1.16	21.72 ± 20.01	2,139 ± 754.3	57.27 ± 3.87	12.91 ± 1.59
*3/*3 (*n* = 22)	1,097 ± 389.1	1.73 ± 0.69	8.50 ± 1.17	7,373 ± 2,185	167.1 ± 80.85	2.59 ± 0.88	22.07 ± 21.76	2,034 ± 767.8	64.68 ± 8.25	13.63 ± 2.09
*P-value*	0.5834	0.5903	0.4412	0.7837	0.7827	0.9443	0.9469	0.7250	0.8130	0.7940
FDR	0.7574	0.6416	0.8050	0.8817	0.9828	0.9443	0.9704	0.7606	0.9326	0.9800
UGT1A1*6										
GG (*n* = 41)	1,120 ± 366.4	1.62 ± 0.69	8.54 ± 1.79	7,017 ± 2,116	171.4 ± 80.34	2.61 ± 0.98	21.70 ± 18.64	2,076 ± 757.4	60.98 ± 4.90	12.67 ± 1.39
GA or AA (*n* = 10)	1,168 ± 381.3	1.90 ± 0.66	8.38 ± 0.78	8,338 ± 2,190	166.2 ± 88.19	2.8 ± 1.32	22.57 ± 28.37	2,163 ± 777.7	58.10 ± 6.66	15.50 ± 3.01
*P-value*	0.7125	0.2533	0.7742	0.0660	0.8774	0.6727	0.7668	0.7131	0.8290	0.2300
FDR	0.7574	0.5803	0.8824	0.4887	0.9828	0.8837	0.9704	0.7606	0.9326	0.7380
UGT1A1*28										
(TA)6/(TA)6 (*n* = 40)	1,162 ± 365.6	1.73 ± 0.70	8.65 ± 1.73	7,533 ± 2,147	174.6 ± 84.60	2.63 ± 0.99	22.05 ± 21.63	2,108 ± 705.2	63.54 ± 5.21	13.76 ± 1.48
(TA)7/(TA)6 or (TA)7/(TA)7 (*n* = 11)	1,012 ± 359.5	1.50 ± 0.63	8.00 ± 1.16	6,343 ± 2,104	155.0 ± 67.80	2.73 ± 1.25	21.22 ± 17.02	2,040 ± 949.5	50.24 ± 3.35	11.41 ± 2.44
*P-value*	0.2341	0.3392	0.2413	0.1086	0.5592	0.7761	0.9066	0.4707	0.3030	0.6030
FDR	0.7574	0.6106	0.7239	0.4887	0.9828	0.8837	0.9704	0.7606	0.8235	0.9800
UGT2B7*2										
GG (*n* = 25)	1,136 ± 395.7	1.74 ± 0.78	8.72 ± 2.05	7,156 ± 2,222	183.5 ± 91.46	2.44 ± 1.02	16.22 ± 11.04	1,967 ± 675.1	56.54 ± 5.88	12.95 ± 1.96
GA (*n* = 20)	1,163 ± 362.5	1.68 ± 0.63	8.25 ± 1.00	7,810 ± 2,117	169.8 ± 70.90	2.70 ± 0.71	21.76 ± 21.20	2,298 ± 885.5	66.84 ± 7.32	13.97 ± 1.97
AA (*n* = 6)	989.2 ± 245.6	1.42 ± 0.38	8.51 ± 1.51	6,000 ± 1,832	117.3 ± 46.75	3.33 ± 1.75	45.79 ± 33.04	1,937 ± 496.0	54.36 ± 6.72	11.75 ± 3.21
*P-value*	0.5982	0.6013	0.8116	0.2032	0.1191	0.1541	0.1289	0.3501	0.3100	0.8960
FDR	0.7574	0.6416	0.8824	0.6096	0.3573	0.6320	0.4020	0.7606	0.8235	0.9800
UGT2B7*3										
GG (*n* = 38)	1,100 ± 350.8	1.71 ± 0.64	8.42 ± 1.15	7,152 ± 2,166	169.0 ± 77.15	2.65 ± 0.99	22.47 ± 22.55	2,141 ± 785.9	61.39 ± 4.86	12.99 ± 1.44
GT or TT (*n* = 13)	1,215 ± 410.0	1.58 ± 0.81	8.77 ± 2.63	7,638 ± 2,242	174.3 ± 94.83	2.65 ± 1.21	20.11 ± 13.78	1,954 ± 662.0	57.27 ± 8.01	13.95 ± 2.76
*P-value*	0.3355	0.4343	0.6577	0.5379	0.9828	0.7855	0.8712	0.4460	0.6880	0.9800
FDR	0.7574	0.6416	0.8824	0.6916	0.9828	0.8837	0.9704	0.7606	0.9326	0.9800
***SLCO1B1*** 521T>C										
TT (*n* = 37)	1,104 ± 338.8	1.65 ± 0.73	8.53 ± 1.79	7,153 ± 2,059	159.7 ± 72.12	2.74 ± 1.12	24.54 ± 23.05	2,054 ± 750.4	59.44 ± 5.06	14.65 ± 1.54
TC (*n* = 14)	1,195 ± 436.9	1.75 ± 0.58	8.46 ± 1.18	7,602 ± 2,505	198.4 ± 98.46	2.39 ± 0.76	14.83 ± 9.06	2,199 ± 783.0	63.00 ± 7.23	9.46 ± 1.84
*P-value*	0.5335	0.6416	0.8824	0.5154	0.2130	0.2878	0.1340	0.4164	0.2740	0.1570
FDR	0.7574	0.6416	0.8824	0.6916	0.4793	0.6320	0.4020	0.7606	0.8235	0.7380
***SLCO1B1*** 388A>G										
GG (*n* = 29)	1,201 ± 357.0	1.78 ± 0.70	8.19 ± 1.06	7,559 ± 2,163	193.0 ± 90.90	2.47 ± 0.73	18.32 ± 18.77	2,212 ± 846.0	62.27 ± 5.40	13.61 ± 1.67
GA or AA (*n* = 21)	1,051 ± 364.7	1.55 ± 0.65	8.94 ± 2.13	7,013 ± 2,172	138.8 ± 55.35	2.93 ± 1.34	26.56 ± 22.29	1,959 ± 602.0	57.93 ± 6.55	12.68 ± 1.98
*P-value*	0.1518	0.209	0.2346	0.3839	0.0152	0.2796	0.0373	0.2464	0.3660	0.4870
FDR	0.7574	0.5803	0.7239	0.6916	0.1368	0.6320	0.3357	0.7606	0.8235	0.9800

*Data of pharmacokinetcs and recovery of platelet function are respectively presented as mean ± SD and mean ± SE, statistical significance for the association of candidate SNPs with pharmacokinetics was analyzed using one-way ANOVA or independent-samples t-test; the association of candidate SNPs with the recovery of platelet function was analyzed using Mann-Whitney U-test or Kruskal-Wallis test*.

† *RT_50_ and RECDAY were successfully acquired in 47 subjects. Abbreviations as in Table 1. The color values refer to the results with P < 0.05 that considered as significant*.

### Effects of Genotypes on Recovery of Platelet Function

Table 3 also shows the associations among the *CYP2C19, CYP3A5, UGT1A1, UGT2B7*, and *SLCO1B1* genotypes and the recovery of platelet function. This finding was genetically attempted to explain the remarkable inter-variabilities of RT_50_ and RECDAY. Although CYP2C19 and SLCO1B1 genetic variants were related to the pharmacokinetics of ticagrelor and M8, no statistically significant difference was observed in the recovery of the platelet activity of these candidate SNPs (*P* > 0.05).

## Discussion

The sufficient recovery of platelet function is essential for patients who need urgent surgery, such as CABG, to reduce the incidence of bleeding. However, the pharmacokinetic parameters and recovery time of the of platelet function showed considerable interindividual variability in 51 healthy Chinese subjects after they received a single dose of ticagrelor. We first identified that t_1/2_ of M8, which exhibited the largest interindividual variability, was significantly associated with RT_50_, implying that the elimination rate of the major active metabolite of ticagrelor is an important factor that causes a remarkable interindividual variability of the recovery of platelet function. CYP2C19^*^3 A allele was related to decreased T_max_ of ticagrelor and the A allele of SLCO1B1 388A>G and CYP2C19^*^2 was associated with decreased C_max_ of M8. Whereas, those nine SNPs were not observed to influence the recovery of platelet function.

The PK parameters of ticagrelor and M8 in these Chinese healthy volunteers was similar to those previously published on healthy Chinese volunteers (Li et al., 2012). Ticagrelor was rapidly absorbed and had a rapid onset of antiplatelet action; this finding was consistent with a previous study on healthy subjects (Teng and Butler, 2010; Jeon et al., 2015), because ticagrelor binds rapidly, potently, and reversibly to the P2Y12 receptor, and acts directly without metabolic activation (James et al., 2009; Teng et al., 2010; Wiviott and Steg, 2015). Although ticagrelor does not require metabolic activation to induce the antiplatelet activity, it is extensively metabolized into the active metabolite M8, which inhibits the P2Y12 receptor at equal potency and exists at ~30–40% of the plasma concentration of ticagrelor (Husted et al., 2006; Zeng et al., 2009; Teng et al., 2010). The characteristics of the ticagrelor-mediated inhibition of platelet aggregation (IPA) are dose related, and gradually declines as the plasma concentrations of active substances decrease (Tantry et al., 2007; Teng and Butler, 2010; Hiasa et al., 2014). When the drug activity is linearly dependent on its plasma concentration, the elimination t_1/2_, which is directly correlated with biological t_1/2_, can be a predictor of the duration of drug pharmacological effects. Therefore, the elimination t_1/2_ of ticagrelor and M8 may be one of the predominant determinants of the recovery time of platelet function after drugs are administered. In this study, t_1/2_ of M8 was first demonstrated to be significantly associated with RT_50_, and both of them had a large individual variation. Therefore, the pharmacokinetics of ticagrelor, particularly the elimination of M8, should be critically considered in the individualized timing of stopping ticagrelor before coronary surgery is performed to ensure the sufficient recovery of platelet function, consequently improving efficacy and reducing bleeding risks.

Previous research found that only three different genetic loci, namely, *SLCO1B1, CYP3A4*, and *UGT2B7*, are associated with ticagrelor levels (Varenhorst et al., 2015). M8 is formed via *CYP3A4* and *CYP3A5* (Zhou et al., 2011), which is the most abundant hepatic CYP enzyme. ~30–90% individual variability in *CYP3A* activity is attributed to genetic variants (Hu et al., 2006; Agrawal et al., 2010), where CYP3A4^*^1G and CYP3A5^*^3 are particularly important because of high frequency in Chinese. CYP3A4^*^1G is in strong linkage disequilibrium with CYP3A5^*^3 (Fukushima-Uesaka et al., 2004). However, the CYP3A4^*^1G polymorphism significantly associated with the pharmacokinetics of M8 did not influence the IPA (Liu et al., 2017), and the platelet function in response to ticagrelor also is not affected by *CYP2C19, ABCB1, P2RY12, P2RY1*, and *ITGB3* genotypes (Storey et al., 2009; Tantry et al., 2010; Wallentin et al., 2010). To investigate the influence of genetic variants related to the ticagrelor pharmacokinetics on the variability of the recovery of the platelet function in Chinese volunteers after they received a single dose of ticagrelor, we selected candidate SNPs in *CYP2C19, CYP3A5, UGT1A1, UGT2B7*, and *SLCO1B1*, which exhibited a relatively high variant allele frequency in Chinese; these SNPs were also documented in pharmacokinetics (Jada et al., 2007; Kim et al., 2008; Wallentin et al., 2010; Wang et al., 2012; Lu et al., 2015; Varenhorst et al., 2015).

CYP2C19^*^2 A was significantly related to decreased C_max_. T_max_ of ticagrelor for CYP2C19^*^3 wildtypes was significantly higher than heterozygous mutations in the study. CYP2C19^*^2 and CYP2C19^*^3 appeared to be among the most important alleles in Chinese. A vitro experiment for the evaluation of enzymes responsible for the metabolism of ticagrelor showed that small amounts of M8 were detected in the incubations with CYP2C19, which indicates CYP2C19 may has some effects on the metabolism of ticagrelor to M8 (Zhou et al., 2011). The incidence of the common variant allele of CYP2C19, such as CYP2C19 ^*^2/^*^2, CYP2C19 ^*^2/^*^3, and CYP2C19 ^*^3/^*^3, which is defined as a slow metabolism type is much higher in Asians (10–25%) than in whites and Africans (Wedlund, 2000). The loss of function in the allele of *CYP2C19* (^*^2 or ^*^3) is associated with the poor antiplatelet activity of clopidogrel and the increased risk of ischemic events (Collet et al., 2009; Mega et al., 2009). However, a PLATO trial has demonstrated that *CYP2C19* genetic polymorphisms have no effect on the efficacy of ticagrelor (Wallentin et al., 2010), and this observation is consistent with our finding.

The current knowledge on ticagrelor absorption is limited, and only P-glycoprotein (P-gp) encoded by *ABCB1*, whose polymorphism exhibits no interaction with ischemia or bleeding, may be involved in ticagrelor absorption from the intestines (Wallentin et al., 2010). *SLCO1B1* 388A>G was associated with decreased C_max_ of M8, but *SLCO1B1* 521T>C did not affect either pharmacokinetic parameters or the recovery of the platelet function to ticagrelor which is inconsistent with previous findings (Varenhorst et al., 2015). *SLCO1B1* encodes a transporter, namely, OATP1B1, which is expressed in human hepatocytes. *SLCO1B1* is responsible for transporting numerous endogenous substances and drugs into the liver for removal (Kalliokoski and Niemi, 2009). Pharmacogenomic research has indicated that SLCO1B1^*^5 can reduce hepatic uptake and increase statin concentrations, leading to an increased risk of simvastatin-induced myopathy (Link et al., 2008; Voora et al., 2009). *SLCO1B1* variants are associated with decreased methotrexate clearance and increased gastrointestinal toxicity for the treatment of acute lymphoblastic leukemia (Ramsey et al., 2012).

However, no candidate SNPs have been observed to influence the recovery of platelet function in these healthy Chinese subjects. This observation agreed with previous findings that showed no genetic determinants for the inhibition of platelets by ticagrelor in published studies (Storey et al., 2009; Tantry et al., 2010; Wallentin et al., 2010) possibly because (i) ticagrelor that was rapidly absorbed and exhibited a rapid onset of antiplatelet responses, thereby leading to the limited effects of these loci on the pharmacokinetics of ticagrelor; (ii) a small sample number was used; and (iii) certain identified genes participated in the metabolism of ticagrelor into M8 with an equipotent antiplatelet activity. Although the clinical application of genetic testing to the improved clinical outcome lacks sufficient evidence, an improved understanding of the genetic determinants of ticagrelor can optimize therapeutic strategies and promote individualized P2Y12 inhibitor treatments based on gene variants. The genetic basis for variance in the recovery of platelet function should be further examined to gain insights into the individualized choice of timing the last dose of ticagrelor before coronary surgery is performed.

After a single dose of ticagrelor (180 mg) was administered, ticagrelor and M8 had t_1/2_ of 8.5 and 21.9 h, whereas the population estimates of RT_50_ were still up to 54.8 h. The mean RECDAY was approximately more than 13 days because of the increased monitoring time until ADP-induced PA reached 69% in our study. However, the complete recovery of platelet function is commonly considered to be 3–5 days after ticagrelor withdrawal (Franchi et al., 2015). All of these observations may be mainly attributed to the large interindividual variation in the recovery time of platelet responses after ticagrelor treatment is discontinued, and this finding was consistent with that of Hansson on patients with ACS (Hansson et al., 2016). At first, the participants in this study were young and healthy, and they generally showed a rapid and strong platelet response, possibly leading to the slow recovery of platelet function after they received a single dose of ticagrelor. The elderly subjects with highest ticagrelor exposure tend to have lower IPA than younger subjects (Teng et al., 2012). In addition, ethnicity and disease status may also lead to differences in the inhibition of the platelet function of ticagrelor. Based on previous study, we observed that Japanese healthy subjects had a lower greatest mean IPA than Caucasians, and the patients with atherosclerosis or CAD also showed a lower greatest mean IPA than the healthy volunteers (Husted et al., 2006; Teng and Butler, 2010, 2014; Hiasa et al., 2014). Therefore, the optimal time point of stopping ticagrelor should be evaluated and determined based on the actual condition of the recovery of platelet function in every patient awaiting CABG rather than simply according to the recommendation (Held et al., 2011; Varenhorst et al., 2012) of withholding ticagrelor for 24–72 h or shortening interval to 2–3 days, which was safer.

Several limitations merit attention. (1) The sample size was small, which may limit the power to identify the genetic variants. However, this is a rigorous full-time-point pharmacokinetic–pharmacodynamic study. Confounding factors such as age, diet and sex ratio were well controlled, and coagulation function, blood routine examination, liver and function, heart rate and electrocardiogram were closely monitored, thereby allow us to clearly and robustly reveal the pharmacokinetics and pharmacogenetic factors that contribute to the recovery of platelet function. (2) Only single-dose oral ticagrelor in healthy subjects was used to study platelet function recovery after the last dose of ticagrelor was administered. In addition to ensuring security and operability, this approach also allowing us to efficiently deploy a rigorous full-time-point PK–PD study. Further studies should also be conducted with multiple ticagrelor doses in patients, which have greater feasibility.

## Conclusions

This study suggests that the M8 elimination is an important factor in determining the recovery of platelet function. The optimal time point of stopping ticagrelor before CABG should be considered individually based on the pharmacokinetics of M8 as well as the actual condition of platelet function recovery. Although CYP2C19 and SLCO1B1 genetic variants were related to the pharmacokinetics of ticagrelor or M8, whereas they were not observed to influence the recovery of platelet function. Further studies should be conducted to enhance our understanding of the genetic determinants of ticagrelor in the recovery of platelet function.

## Ethics Statement

This study was approved by the Medical Ethical Review Committee of Guangdong General Hospital and conducted according to the Declaration of Helsinki.

## Author Contributions

QZ, WZ, and XW performed experiment, performed data analysis, and wrote the manuscript. LM and GH participated in patient recruitment and performed experiment. JC, LT, and SL revised manuscript. SZ and WL designed the study and revised manuscript. All authors reviewed and approved the final manuscript.

### Conflict of Interest Statement

The authors declare that the research was conducted in the absence of any commercial or financial relationships that could be construed as a potential conflict of interest.
