# Supplementary material for: Pharmacokinetic and Pharmacogenetic Factors Contributing to Platelet Function Recovery After Single Dose of Ticagrelor in Healthy Subjects
Source: Front Pharmacol. 2019 Mar 18;10:209. doi: 10.3389/fphar.2019.00209 (PMC6431676; doi:10.3389/fphar.2019.00209)
Supplement: Supplementary file 1 [file Table_1.DOCX]

***Supplementary Material***

**Pharmacokinetics and pharmacogenetic factors contributing to platelet function** **recovery after single dose of ticagrelor in healthy subjects**

**Qian Zhu^1,2,3†^, Wan-Ping Zhong^1,2†^, Xi-Pei Wang^1†^, Li-Ping Mai^2^, Guo-Dong He^2^, Ji-Yan Chen^1^, Lan Tang^3^, Shu-Wen Liu^3^, Wei-Hua Lai^2*^, Shi-Long Zhong ^1,2, 3*^**

^†^ These authors contributed equally to the study.

***Correspondence:**

Shi-Long Zhong: [zhongsl@hotmail.com](mailto:zhongsl@hotmail.com)

Wei-Hua Lai: [laiweihuax@163.com](mailto:laiweihuax@163.com)

**1 Supplementary Methods**

**1.1 Sample preparation for LC-MS/MS analysis**

The plasma samples were thawed at room temperature (RT) before being subjected to extraction. An aliquot of plasma (200 µL) was combined with ferulic acid working solution (10 µg/mL, 5 µL), extracted with ethyl acetate (1.3 mL) and vortex-mixed for 5 min at RT. The sample was centrifuged at 6000 rpm for 10 min at 4°C, and an aliquot (1 mL) of the supernatant was evaporated to dryness under vacuum at ambient temperature. The residue was reconstituted in 50% water/acetonitrile (100 µL). The mixture was vortexed for 3 min at RT, followed by centrifugation at 12,000 rpm for 15 min at 4°C. An aliquot (5 µL) of the supernatant was analyzed by LC-MS/MS.

**1.2 Determination of ticagrelor and its metabolites concentrations**

A high-performance liquid chromatography coupled with tandem mass spectrometry (LC-MS/MS) assay was developed and validated for simultaneous determination of ticagrelor and its metabolites in human plasma. The system consisted of a prominence LC-20AB integrated system (Shimadzu, Kyoto, Japan) and an API 4000 QTrap mass spectrometer (AB Sciex, Redwood City, CA, USA) equipped with an electrospray ionization unit.

Chromatographic separation was achieved on an Ultimate XB-C18 column (2.1 mm × 150mm, 3µm) (Welch Materials Inc., Maryland, USA) at ambient temperature. The mobile phase consisted of aqueous ammonium acetate solution (0.025 mM) and acetonitrile (35:65, *v*: *v*) at a flow rate of 0.25 mL/min. The injection volume was 5 µL and the analytical run time was 4.5 min. Declustering potential (DP), entrance potential (EP), and collision energy (CE) were adjusted to give maximum sensitivity in negative ionization mode, respectively. Data acquisition was processed using Analyst 1.4.2 software (AB Sciex). Optimal mass spectrometry parameters for ticagrelor, M8 and internal standard (IS, ferulic acid) are shown in the Table below.

| **Compound** | ***m/z* (M-H)^-^** | **DP** (V) | **EP** (V) | **CE** (V) | **Retention time** (min) |
| --- | --- | --- | --- | --- | --- |
| Ticagrelor | 521.2→361.2 | -140 | -9 | -32 | 3.50 |
| M8 | 477.2→361.1 | -130 | -9 | -30 | 3.41 |
| IS | 193.0→133.7 | -56 | -10 | -23 | 1.64 |

**1.3 Platelet function testing**

Platelet aggregation (PA) was measured using a Chrono-log Platelet Aggregation Systems (VASTEC MED. Ltd.) as follows: whole blood was centrifuged at 1000 rpm for 10 min and the platelet-rich plasma (PRP) collected, then platelet-poor plasma (PPP) prepared by further centrifugation at 3000 rpm for 20 min. PPP and PRP (adjusted to 100×10^9^/L) were used as 100% and 0% references without agonists, respectively. The PA post-dose till recovery to baseline was measured by light transmission method using ADP (20 μmol/L final concentration), and expressed as percentage.

**2 Supplementary Tables**

**Table S1** Sequences of the primers and probes used for genotyping

| **SNPs** |  | **Primers/Probes (5’ to 3’)** |
| --- | --- | --- |
| ***CYP2C19*2*** | forward | ATTACAACCAGAGCTTGGCATATTG |
|  | reverse | CGATTCTTGGTGTTCTTTTACTTTCTC |
|  | Wt | HEX-ATTATTTCCCGGGAACC-MGB |
|  | Mt | FAM-ATTATTTCCCAGGAACC-MGB |
| ***CYP2C19*3*** | forward | GATCAGCAATTTCTTAACTTGATGGA |
|  | reverse | ACTGTAAGTGGTTTCTCAGGAAGCA |
|  | Wt | HEX-ATTGTAAGCACCCCCTGGATCCAG-BHQ1 |
|  | Mt | FAM-TTGTAAGCACCCCCTGAATCCAGG-BHQ1 |
| ***CYP3A5*3*** | forward | AACATTATGGAGAGTGGCATAGGAG |
|  | reverse | TGTAATCCATACCCCTAGTTGTACGA |
|  | Wt | FAM-TGTCTTTCAATATCTCTTC-MGB |
|  | Mt | HEX-TTTTGTCTTTCAGTATCTC-MGB |
| ***UGT1A1*6*** | forward | CTAGCACCTGACGCCTCGTT |
|  | reverse | CTCTTTCACATCCTCCCTTTGG |
|  | Wt | FAM-CATCAGAGACAGAGCA-MGB |
|  | Mt | HEX-CATCAGAGACGGAGCA-MGB |
| ***UGT1A1*28*** | forward | AACATTAACTTGGTGTATCGATTGGT |
|  | reverse | AGCAGGCCCAGGACAAGT |
|  | Wt | FAM-TTGCCATATATATATATATAAGTAGGA-MGB |
|  | Mt | VIC-TGCCATATATATATATATATAAGTAGGA-MGB |
| ***UGT2B7*2*** | forward | GGGTTTGGCAGGTTTGCA |
|  | reverse | GGCTTATTCGAAACTCCTGGAA |
|  | Wt | 7VIC-AGTGGATATGGAAAC-MGBNFQ |
|  | Mt | 6FAM-TAAGAGTGGATGTGGAAAC-MGBNFQ |
| ***UGT2B7*3*** | forward | TCTTCAGCTTCCATTCTTTTTGATC |
|  | reverse | GACCATCTCTTAATCTGTTGCATGA |
|  | Wt | 7VIC-AACAACTCATCCTCTCT-MGBNFQ |
|  | Mt | 6FAM-CAACAACTCATCCGCT-MGBNFQ |
| ***SLCO1B1*5*** | forward | TCCCCTATTCCACGAAGCAT |
|  | reverse | GGTTGTTTAAAGGAATCTGGGTCA |
|  | Wt | HEX-TTACCCATGAACACATAT-MGB |
|  | Mt | FAM-TACCCATGAACGCATAT-MGB |
| ***SLCO1B1*1b*** | forward | TCAGTGATGTTCTTACAGTTACAGGTATTCT |
|  | reverse | ATCTCAGGTGATGCTCTATTGAGTGA |
|  | Wt | HEX-AAGAAACTAATATCGATTCAT-MGB |
|  | Mt | FAM-AAGAAACTAATATCAATTCAT-MGB |
